# Early and late recurrent cardiovascular events among high‐risk patients with an acute coronary syndrome: Meta‐analysis of phase III studies and implications on trial design

**DOI:** 10.1002/clc.23773

**Published:** 2022-01-12

**Authors:** Gerald Chi, Jane J. Lee, Syed H. A. Kazmi, Clara Fitzgerald, Sahar Memar Montazerin, Arzu Kalayci, Serge Korjian, Mark Heise, Lawrence I. Deckelbaum, Peter Libby, Deepak L. Bhatt, C. Michael Gibson

**Affiliations:** ^1^ Department of Medicine, Division of Cardiovascular Medicine, Beth Israel Deaconess Medical Center Harvard Medical School Boston Massachusetts USA; ^2^ Baim Institute for Clinical Research Boston Massachusetts USA; ^3^ CSL Behring King of Prussia Pennsylvania USA; ^4^ Division of Cardiovascular Medicine, Brigham and Women's Hospital Harvard Medical School Boston Massachusetts USA

**Keywords:** acute coronary syndrome, atherosclerosis, coronary artery disease, secondary prevention, thrombosis

## Abstract

**Background:**

Despite low‐density lipoprotein cholesterol‐lowering therapies and other standard‐of‐care therapy, there remains a substantial residual atherosclerotic risk among patients with an acute coronary syndrome (ACS). This study aims to estimate the risk of early and late recurrent major adverse cardiovascular events (MACE) and address its implications on trial design.

**Methods:**

A literature search was performed to collect phase III interventional trials on high‐risk ACS patients. Pooled event rates at 90 and 360 days were estimated by fitting random‐effects models using the DerSimonian–Laird method. Under the assumption of a total sample size of 10,000 and 1:1 allocation at a one‐sided alpha of 0.025 using the log‐rank test, the relationship between power and relative risk reduction (RRR) or absolute risk reduction (ARR) was explored for early versus late MACE endpoint.

**Results:**

Seven trials representing 82,727 recent ACS patients were analyzed. The pooled rates of recurrent MACE were 4.1% and 8.3% at 90 and 360 days. Approximately 49% of events occurred within the first 90 days. Based on the estimated risks at 90 and 360 days, to attain 90% statistical power, a lower magnitude of RRR is required for late MACE than early MACE (22% vs. 30%), whereas a lower magnitude of ARR is required for early MACE than late MACE (1.2% vs. 1.8%).

**Conclusion:**

The initial 90‐day window after ACS represents a vulnerable period for recurrent events. From a trial design perspective, determining a clinically important benefit by RRR versus ARR may influence the decision between early and late MACE as the study endpoint.

## 1 INTRODUCTION

Current practice guidelines recommend the use of statins for secondary prevention following acute coronary syndrome (ACS).^1^ Yet, substantial risk for recurrent atherosclerotic events exists among individuals who achieve low‐density lipoprotein cholesterol (LDL‐C) goals. The residual risk remains approximately 9% per year among patients with established coronary artery disease (CAD) despite aggressive LDL‐C reduction, and may be mediated at least in part by atherogenic triglyceride‐rich lipoproteins and high‐density lipoprotein cholesterol (HDL‐C).^2^ Reducing this residual cardiovascular risk among patients with ACS remains a major unmet medical need. The initial 2‐to‐3‐month period following an ACS event has been recognized as the acute recovery phase characterized by an increased risk of recurrent ischemia, after which the clinical course is similar to chronic stable coronary disease and the risk of recurrent cardiovascular events declines.^3^

The primary aim of the study was to estimate the early (at 90 days) versus late (at 360 days) risk of major adverse cardiovascular events (MACE) from recent landmark cardiovascular trials to better characterize the time course of the residual risk among patients who receive standard‐of‐care therapies following ACS. Furthermore, the incidence of early or late recurrent MACE events and its potential implications with respect to statistical power has never been quantitatively assessed. Accordingly, we also aimed to evaluate the relative statistical power of a primary MACE endpoint at these timepoints under a variety of assumptions. From a trial design standpoint, if most of the recurrent MACE events occur in the early post‐ACS phase, a question naturally arises as to whether setting the primary endpoint at the early phase would yield a greater statistical power compared to setting the primary endpoint at the late phase when a consistent long‐term treatment effect cannot be ascertained. To investigate this question, we compared the statistical power of a primary endpoint at 90 versus 360 days based on the event rates derived from the contemporary phase III randomized controlled trials.

## 2 METHODS

A comprehensive literature search was performed in ClinicalTrials.gov to collect completed phase III interventional trials from inception to June 2019 targeting the study population of high‐risk ACS patients. Full‐text publications of the collected studies were retrieved for screening. Studies fulfilling the following criteria were included: (1) study participants had a recent ACS (up to 90 days from enrollment) and at least one additional cardiovascular risk factor (age ≥ 60 years, diabetes mellitus, multivessel coronary artery disease, polyvascular disease, prior history of myocardial infarction [MI], stroke, or coronary revascularization); (2) study participants received a background of optimal medical therapy including antiplatelet agents (aspirin or P2Y_12_ inhibitor), angiotensin‐converting enzyme inhibitor (ACEI), angiotensin receptor blocker (ARB), beta‐blocker (BB), and statin; (3) the duration of follow‐up was at least 12 months; and (4) the primary efficacy endpoint was MACE, defined as a composite of MI, stroke, or cardiovascular death. Two independent investigators searched and screened the qualified clinical trials, and disagreement was resolved by discussion and consensus. A flow chart depicting the selection of these trials is provided in Figure S1.

Data extracted from each study included study characteristics, intervention, follow‐up duration, patient characteristics, concomitant medications, and MACE event rates at 90 and at 360 days. For studies that did not report MACE risks at 90 or 360 days, the rates were estimated from Kaplan–Meier (KM) curves with a validated data extraction application (DigitizeIt; http://www.digitizeit.de/).^4^ The absolute number of MACE events was estimated by multiplying the KM rate and the number of participants, without accounting for censoring or loss to follow‐up. The relative percentage of early MACE was derived from the ratio of MACE event rate at 90 days to the MACE event rate at 360 days.

The pooled event rates at 90 days and 360 days were estimated with meta‐analytic approaches described as follows. In view of the skewed distribution of observed proportion (i.e., the ratio of the number of events to the number of participants was not centered around 0.5), a logit transformation was performed to conform to the normal distribution. In each study, the natural logarithm of the proportion was deemed as the effect size statistic, and the inverse variance of the transformed proportion was used as study weight. The confidence intervals (CIs) of proportions were calculated by normal approximation. The summary effect size was then computed by fitting a random‐effects model using the DerSimonian and Laird method. Heterogeneity across the studies was assessed using the Q test (with the threshold of *p* < .10 indicating the presence of heterogeneity) and *I* ^2^ statistic (with the values of 0.25, 0.50, and 0.75 indicating a low, moderate, and high degree of heterogeneity, respectively). A sensitivity analysis using the leave‐one‐out approach was performed to test the robustness of estimated event rates at 90 and at 360 days. An independent investigator evaluated the risk of bias of included studies by the Cochrane Collaboration's tool.^5^ The assessment of the risk of bias was validated by a second investigator.

To provide insights on trial design, the study assessed the statistical power of setting the primary endpoint as the early MACE versus late MACE based on estimated event rates pooled from these contemporary phase III trials. Under the assumption of a total sample size of 10,000 and 1:1 allocation at a one‐sided alpha of 0.025 using the log‐rank test, two separate approaches were applied to compare the statistical power associated with early MACE (90 days) versus late MACE (360 days) as the primary efficacy endpoint when prospectively designing an interventional trial. In the first approach, the relationship between power and hazard ratio (ranging from 0.99 to 0.70, corresponding to a relative risk reduction [RRR] from 1% to 30%) was explored. In the second approach, the relationship between power and absolute risk reduction (ARR; ranging from 0.1% to 3.0%) was explored.

The analyses were performed using SAS (version 9.4; SAS Institute Inc.), R software (Version 3.5.2; the R Foundation for Statistical Computing), and Power Analysis and Sample Size (PASS) software (version 11; NCSS LLC).

## 3 RESULTS

### 3.1 Summary of study characteristics

Seven trials, representing a total of 82,727 high‐risk patients with recent ACS, were analyzed (Table S1).^6^, ^7^, ^8^, ^9^, ^10^, ^11^, ^12^ The duration of follow‐up ranged from 12 to 36 months. Various interventions were evaluated, including P2Y_12_ inhibitors in three studies (TRITON‐TIMI 38, PLATO, and TRILOGY ACS), antidiabetic agents in two studies (EXAMINE and AleCardio), factor Xa inhibitor in one study (ATLAS ACS 2‐TIMI 51), and lipoprotein‐associated phospholipase A2 inhibitor in one study (SOLID‐TIMI 52). Most patients received standard of care secondary prevention medications, including aspirin (90.6%–99%), P2Y_12_ inhibitor (79.8%–93.0%), statin (83.1%–94.9%), BB (65.6%–89.7%), and ACEI or ARB (38.2%–88.2%). The majority of the included trials had a low risk of bias (Figure S2).

### 3.2 Absolute risk and relative percentage of early versus late MACE

**Figure 1:**
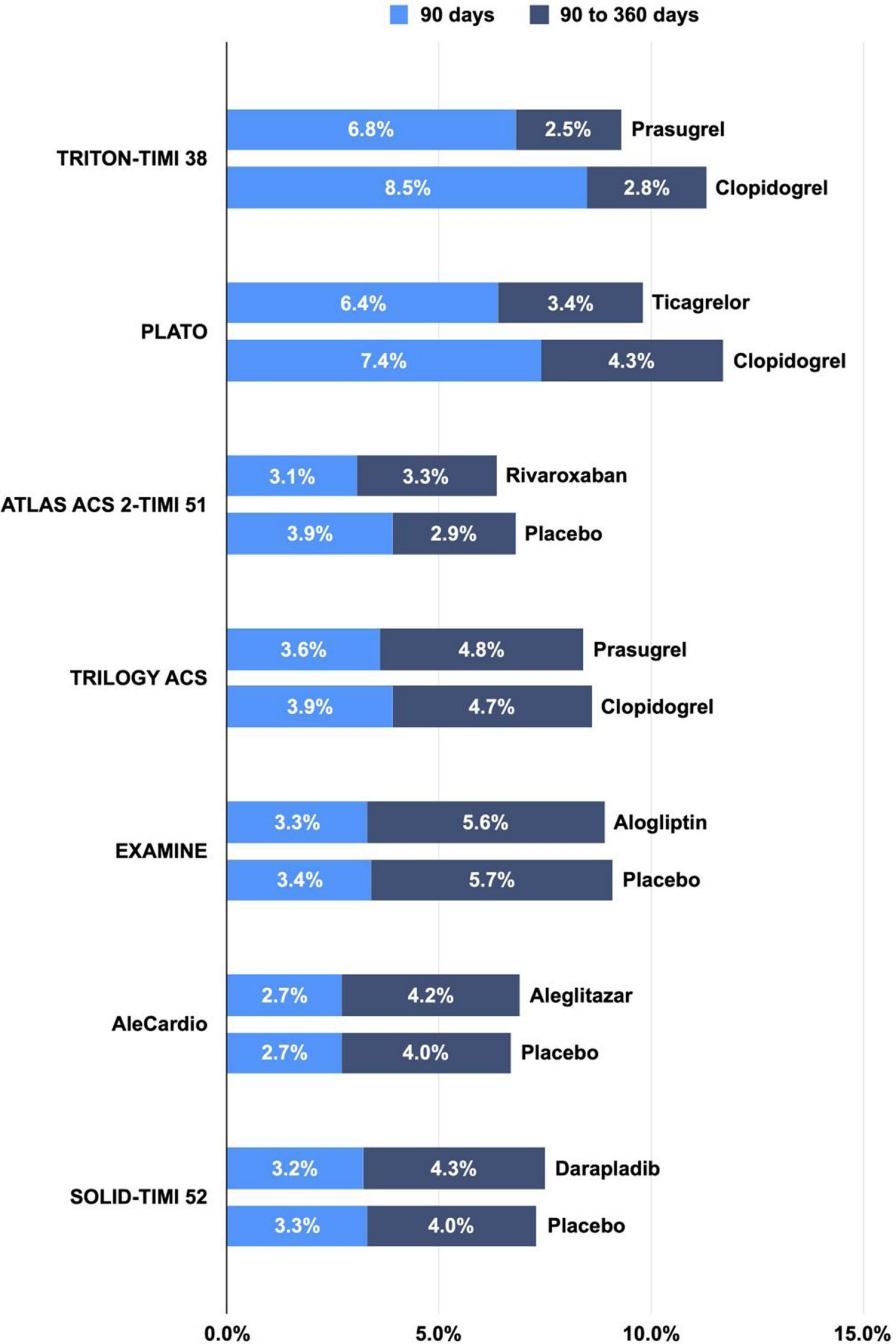
Absolute risk at 90 days versus 90–360 days

**Figure 2:**
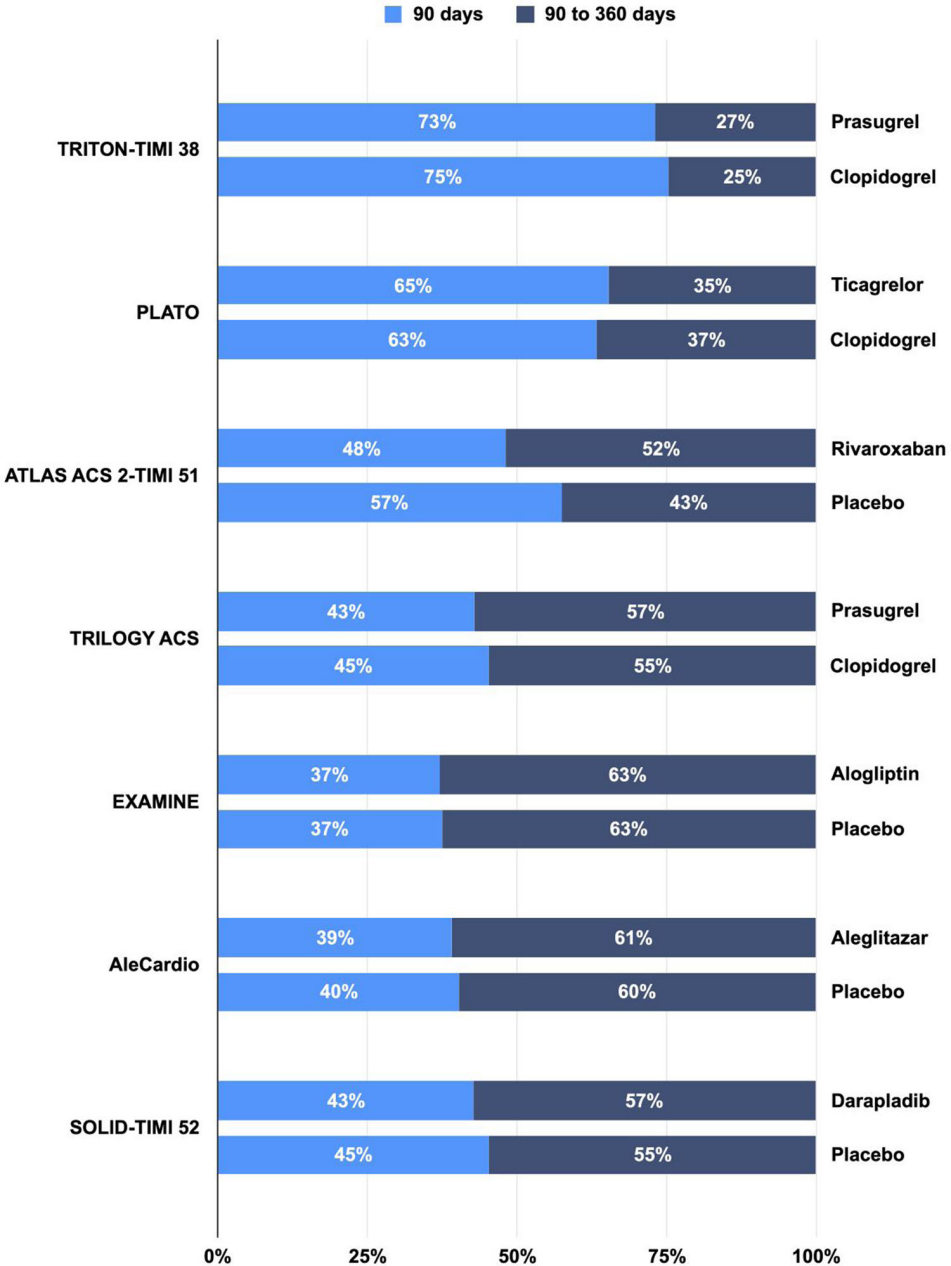
Relative percentage of risk at 90 days versus 90–360 days

**Figure 3:**
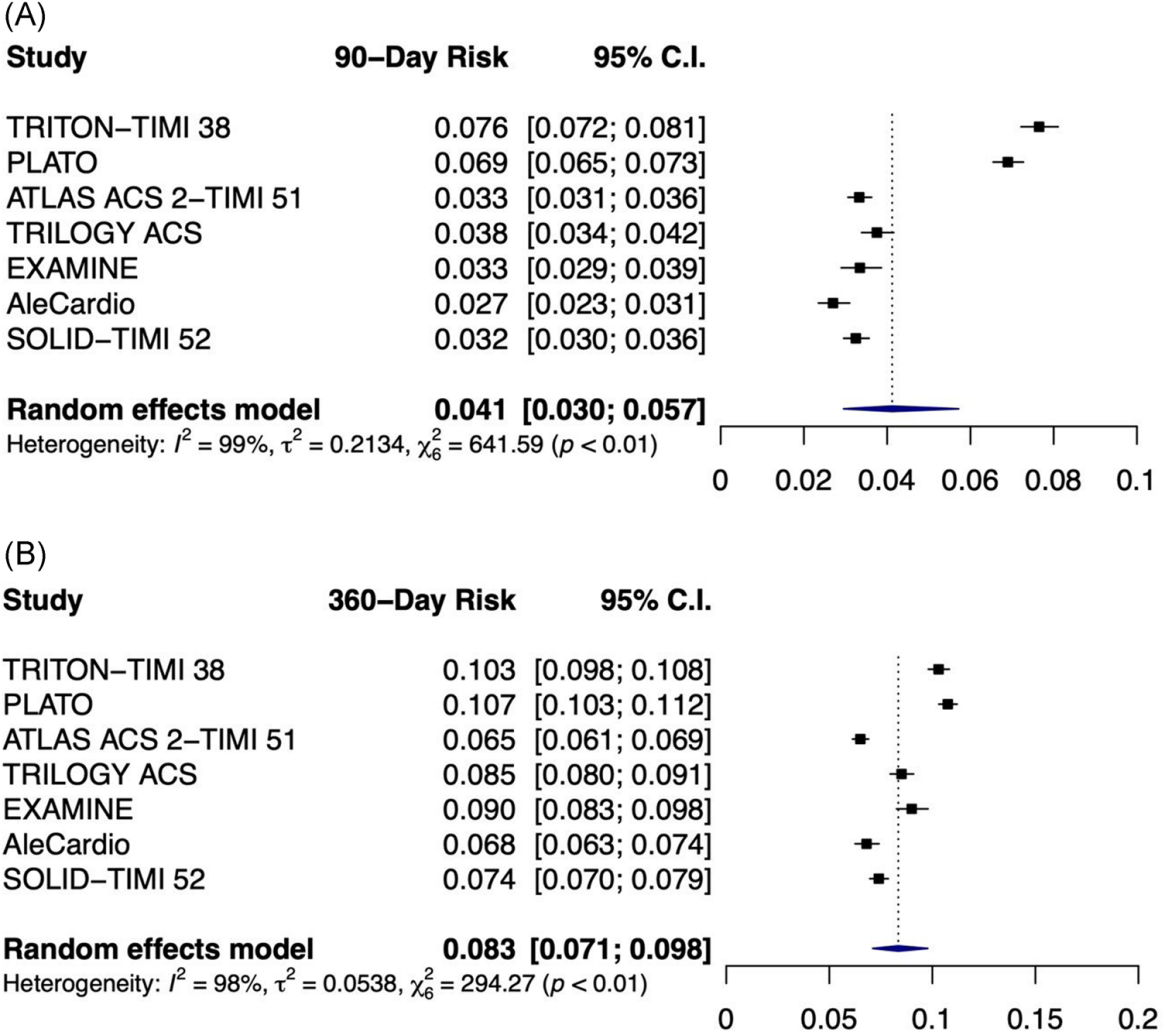
Pooled risk at (A) 90 days and (B) 360 days

The absolute risk of 90‐ and 360‐day MACE ranged from 2.7% to 8.5% and 6.2% to 11.7%, respectively (Table S1 and Figure 1). With respect to the timing of events, 37% to 75% of the MACE events occurred within the first 90 days (Figure 2). Pooled analysis demonstrated that the risk of recurrent MACE was 4.1% at 90 days (95% CI: 3.0%–5.7%; Figures 3A) and 8.3% at 360 days (95% CI: 7.1%–9.8%; Figure 3B), with a high degree of heterogeneity across studies (*p* < .01). Approximately 49% of events occurred within the first 90 days, whereas 51% of events occurred in the subsequent 270 days (between 90 and 360 days). In the sensitivity analysis, the 90‐day risk (ranging from 3.7% to 4.4%; Figure S3) and 360‐day risk (ranging from 8.0% to 8.7%; Figure S4) were similar to the estimates from primary analysis.

### 3.3 Statistical power of early versus late MACE as the primary endpoint

Based on pooled results from contemporary landmark trials, event rates of 4.1% at 90 days and 8.3% at 360 days were assumed in the comparator arm. With a total sample size of 10,000 and 1:1 allocation at a one‐sided alpha of 0.025 using the log‐rank test, two separate approaches were applied to compute the statistical power for early versus late MACE as the primary endpoint when prospectively designing an interventional trial.

### 3.4 Relationship between power and HR

**Figure 4:**
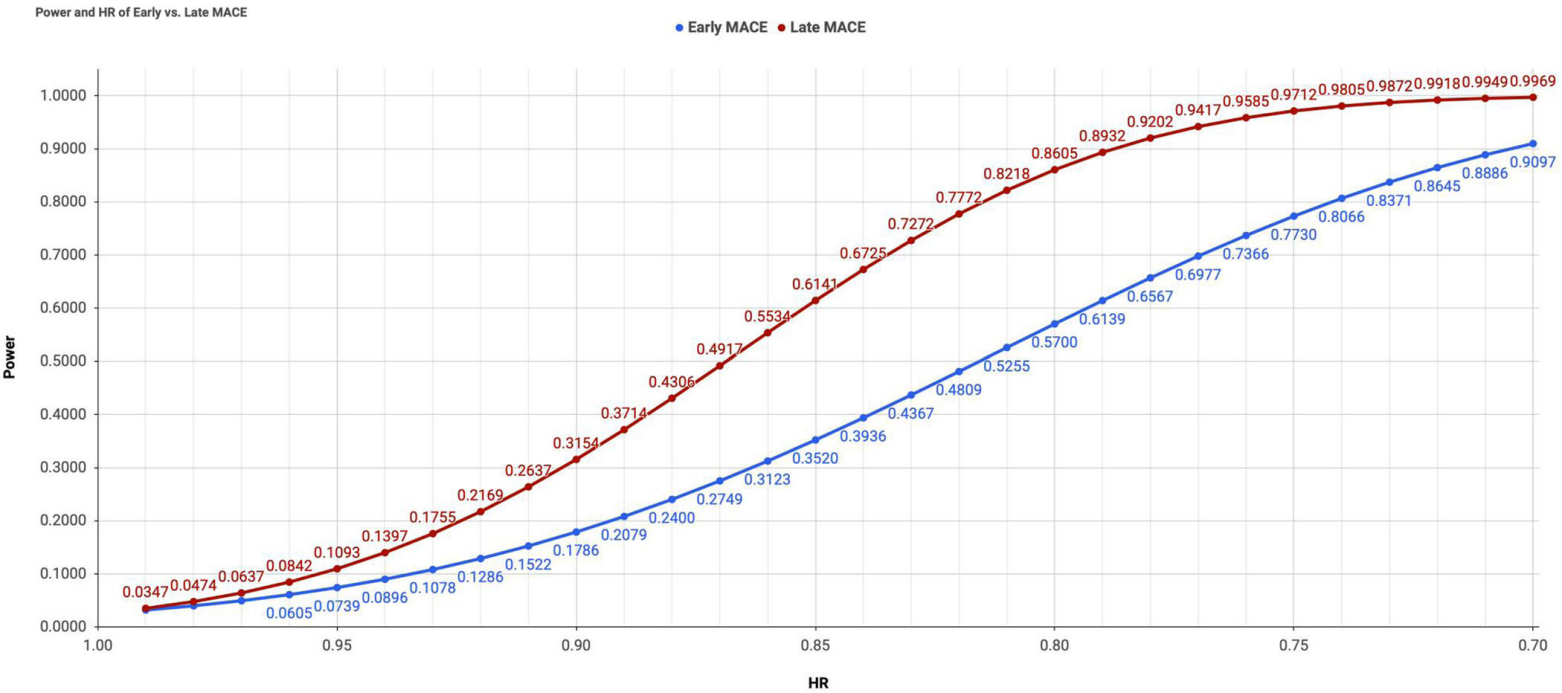
Power and hazard ratio of early versus late major adverse cardiovascular events endpoint

The relationship between power and hazard ratio (ranging from 0.99 to 0.70, corresponding to a RRR from 1% to 30%) was evaluated and is illustrated in Figure 4. When a predetermined HR was employed in the power calculation, greater statistical power was consistently achieved when late MACE was selected as the primary endpoint compared with early MACE. Specifically, with a hazard ratio (HR) of 0.80 (i.e., RRR of 20%) in favor of the intervention, the power would be 86% for the late MACE (corresponding to an event rate of 8.3% in the comparator arm vs. 6.7% in the experimental arm at 360 days, with an ARR of 1.6%) and 57% for early MACE (corresponding to an event rate of 4.1% in the comparator arm vs. 3.3% in the experimental arm at 90 days, with an ARR of 0.8%). Interpreted differently, if RRR were maintained at 20% from 90 to 360 days, there would be an increase in statistical power from 57% to 86%. In this scenario, the KM curves for the experimental and comparator arms may continue to diverge beyond 90 days, reflecting an augmentation of ARR from 0.8% to 1.6%.

If a prespecified HR were used when designing a trial, a lower magnitude of RRR would be required if the late MACE were selected as the primary endpoint. For instance, to attain at least 90% power, an RRR of 22% would be required for the late MACE endpoint (corresponding to 8.3% vs. 6.5% at 360 days) compared to an RRR of 30% for the early MACE endpoint (corresponding to 4.1% vs. 2.9% at 90 days).

### 3.5 Relationship between power and ARR

**Figure 5:**
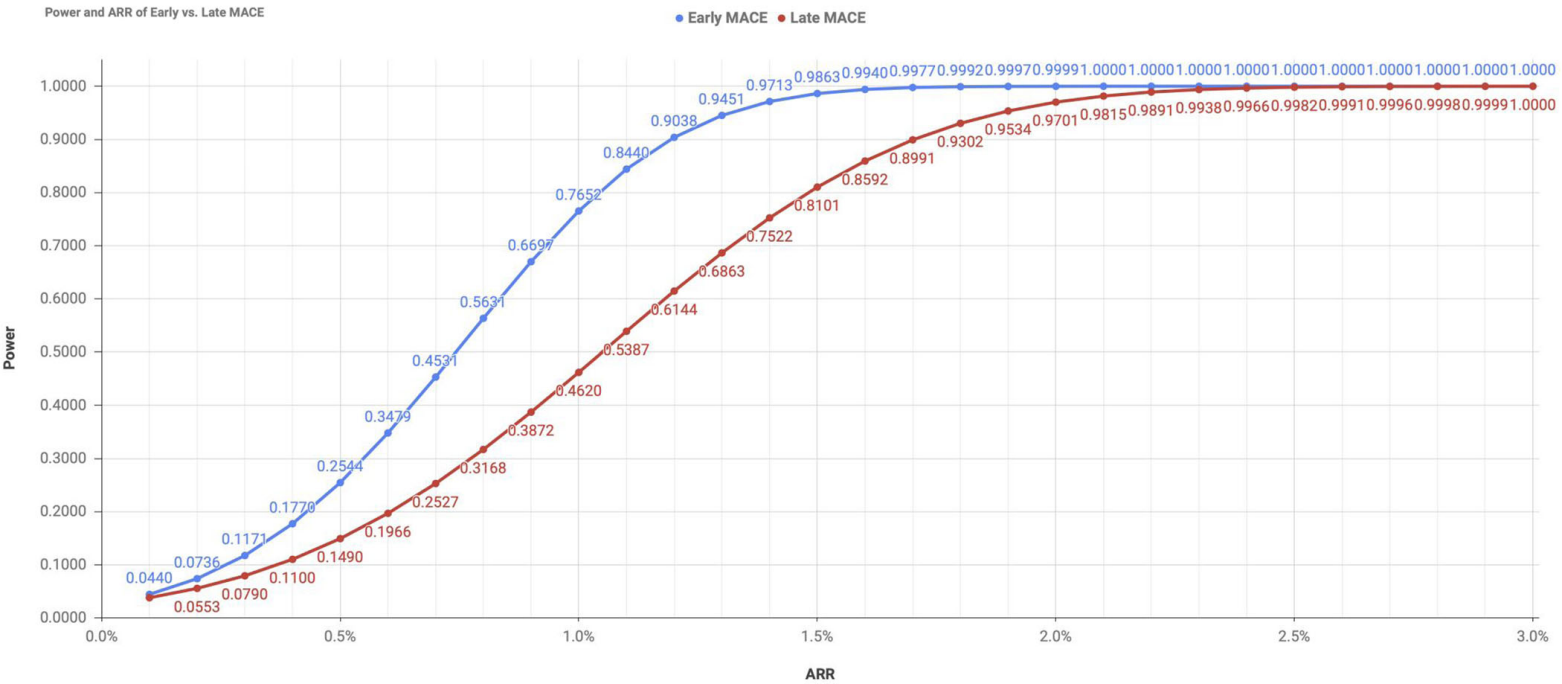
Power and absolute risk reduction of early versus late major adverse cardiovascular events endpoint

The relationship between power and ARR (ranging from 0.1% to 3.0%) was also evaluated and is illustrated in Figure 5. When a predetermined ARR was employed in the power calculation, greater statistical power was consistently achieved when early MACE was selected as the primary endpoint compared with late MACE. Specifically, with an ARR of 1% in favor of the intervention, the power would be 77% for early MACE (corresponding to an event rate of 4.1% in the comparator arm vs. 3.1% in the experimental arm at 90 days, with a hazard ratio [HR] of 0.75) and 46% for late MACE (corresponding to an event rate of 8.3% in the comparator arm vs. 7.3% in the experimental arm at 360 days, with an HR of 0.87). Stated differently, if ARR were maintained at 1.0% from 90 to 360 days, there would be a decline in the statistical power from 77% to 46%. In this scenario, the KM curves for the experimental and comparator arms would be parallel beyond 90 days, which in fact suggests a diminution of RRR from 25% to 13%.

If a prespecified ARR were used when designing a trial, a lower magnitude of ARR would be required if the early MACE were selected as the primary endpoint. For instance, to attain at least 90% power, an ARR of 1.2% would be required for the early MACE endpoint (corresponding to 4.1% vs. 2.9% at 90 days) compared to an ARR of 1.8% for the late MACE endpoint (corresponding to 8.3% vs. 6.5% at 360 days).

## 4 DISCUSSION

The pooled results from contemporary phase III trials of high‐risk ACS patients demonstrate that 4.1% and 8.3% of patients developed a recurrent major cardiovascular event (i.e., cardiovascular death, MI, or stroke) at 90 days and 360 days, respectively. Approximately 49% of events occurred within the first 90 days, whereas 51% of events occur between 90 and 360 days. Therefore, the time course of residual atherosclerotic risk likely follows a nonlinear trajectory, with a steeper slope in the first 90 days, as opposed to the period after 90 days. Despite standard‐of‐care medications, the initial 90 days following an ACS episode should be considered as a vulnerable window for recurrent events. This observation supports the hypothesis of two distinct periods following ACS with an early vulnerable period with markedly heightened cardiovascular risk and a late stable period. This distinction is important when gauging potential treatment benefits in novel agents and when investigating the underlying pathophysiology for recurrent ACS.

The nonlinear trajectory of residual risk was also evident in the meta‐analysis of statin trials from the Cholesterol Treatment Trialists' (CTT) Collaboration.^13^ Specifically, the annual risk of major vascular events was greater in the first year (2.2% in the statin arm vs. 2.5% in the control arm) than in the subsequent 4 years (1.5%–1.6% in the statin arm vs. 1.8%–2.0% in the control arm). More importantly, the magnitude of RRR by statins was less pronounced in the first year (12%) than in the subsequent 4 years (22%–28%). The unattenuated early residual risk points to the unmet medical need for interventions that target atherothrombotic pathways unrelated to platelet activation or LDL‐C (e.g., triglycerides, very‐low‐density lipoproteins [VLDL], and HDL‐C), which have been extensively investigated in the past three decades. For instance, among statin‐treated patients from the PROVE IT‐TIMI 22 trial, a low triglyceride level was associated with a reduced cardiovascular risk independent of LDL‐C levels.^14^ Similarly, pharmacological reductions in the small, triglyceride‐depleted VLDL were associated with a decreased atherosclerotic risk among participants in the JUPITER trial who had low LDL‐C levels.^15^ With respect to HDL‐C, its inverse relationship with cardiovascular disease was supported by a meta‐analysis of 20 statin trials with adjustment for statin potency and LDL‐C levels.^16^ However, HDL‐C‐raising therapies, such as fibrates, niacin, and cholesteryl ester transfer protein inhibitors, have not consistently demonstrated cardiovascular benefits.^17^ Furthermore, Mendelian randomization studies did not demonstrate a causal relationship between genetically‐altered plasma HDL‐C levels and cardiovascular risk.^18^, ^19^, ^20^, ^21^, ^22^ More research is warranted to unravel the underlying mechanisms and optimal preventive strategies for modifying the residual risk.

The first 90 days following an ACS episode marks not only the vulnerable window in which half of the MACE events occur, but also the acute recovery period during which a robust treatment effect could be established. Among the three trials that demonstrated significant efficacy of investigational drugs (i.e., TRITON‐TIMI 38, PLATO, and ATLAS ACS 2‐TIMI 51), a considerable proportion of MACE reduction was attained early at 90 days (TRITON‐TIMI 38: 1.7% at 90 days vs. 2.0% at 360; PLATO: 1.0% at 90 days vs. 1.9% at 360 days, ATLAS ACS 2‐TIMI 51: 0.9% and 0.8% at 90 days vs. 0.6% and 0.3% at 360 days). It is also noteworthy that the treatment effect attained at 90 days was either sustained, expanded, or diminished at 360 days, indicating the need to consider the appropriateness of the Cox proportional hazards model assumption in clinical trial design, as well as the importance of applying alternative approaches to assessing the treatment effect, such as restricted mean survival time.^23^

The distinction of early residual risk has practical implications on trial design. If a novel intervention is hypothesized to primarily reduce early residual risk but not late residual risk (in which Kaplan‐Meier curves separate before 90 days and stay parallel through 360 days), then the early MACE endpoint should be employed for sample size determination, because the early vulnerable period represents a more appropriate window for examining the mechanistic effect of such intervention. In this scenario, the proportionality of hazards assumption may be violated when the primary endpoint is set at 360 days. Conversely, if a novel intervention is hypothesized to reduce both early and residual risk with a constant HR over time, then the late MACE endpoint should be employed for sample size determination, as it accounts for both early and late events. Additional considerations should be made regarding the statistical power. This study demonstrates that the early MACE endpoint is associated with a greater power when ARR is employed to gauge the treatment effect, whereas the late MACE endpoint is associated with a greater power when RRR is employed to gauge the treatment effect. Therefore, a clinically important reduction in ARR or RRR should be predetermined, as the decision affects the statistical power and selection between early versus late MACE endpoint.

## 5 LIMITATIONS

The cumulative incidence from the present analysis was estimated indirectly from the KM curves provided in the original publications. As the exact patient‐level event rates at 90 and at 360 days were not available, the results only serve the purposes of hypothesis‐generating and benchmarking for trial design. Furthermore, the timing of the index ACS event and treatment initiation varied across the studies. Given the variety of enrichment risk factors, the cardiovascular risk profile of patients may differ within and across the studies. In addition, the efficacy of specific therapy to prevent cardiovascular events may vary with the time course. These factors may have contributed to the substantial heterogeneity of 90‐ and 360‐day risks observed in the meta‐analysis. Finally, as multiple treatments were used in the ACS patients, it remains uncertain whether the time‐varying treatment effect applies to only lipid‐lowering medications or other therapies.

## 6 CONCLUSION

The initial 90‐day window after an ACS event represents a vulnerable period for recurrent cardiovascular events, possibly attributable to atherothrombosis mechanisms not sufficiently attended by current optimal medical therapy. When designing an interventional trial, a clinically important reduction in absolute risk versus relative risk should be predetermined, as the decision affects the selection between early MACE versus late MACE as the primary endpoint.

## CONFLICT OF INTERESTS

Dr. Chi receives research grant support paid to the Beth Israel Deaconess Medical Center, Harvard Medical School from Bayer, Janssen Scientific Affairs, and CSL Behring. Dr. Heise and Dr. Deckelbaum are employed by CSL Behring. Dr. Gibson receives consultant fees from Portola Pharmaceuticals and reports grants from Angel Medical Corporation and CSL Behring; grants and other support from Bayer Corporation; grants and personal fees from Janssen, Johnson & Johnson, and Portola Pharmaceuticals; and personal fees from The Medicines Company, Boston Clinical Research Institute, Cardiovascular Research Foundation, Eli Lilly, Gilead Sciences Inc, Novo Nordisk, Pfizer, Web MD, UpToDate in Cardiovascular Medicine, Amarin Pharma, Amgen, Arena Pharmaceuticals, Bayer Corporation, Boehringer Ingelheim, Chiesi, Merck & Co, PharmaMar, Sanofi, Somahlution, St Francis Hospital, and Verreseon Corporation. Dr. Libby is an unpaid consultant to, or involved in clinical trials for Amgen, AstraZeneca, Baim Institute, Beren Therapeutics, Esperion Therapeutics, Genentech, Kancera, Kowa Pharmaceuticals, Medimmune, Merck, Norvo Nordisk, Novartis, Pfizer, and Sanofi‐Regeneron. Dr. Libby is a member of the scientific advisory board for Amgen, Caristo, Cartesian, CSL Behring, DalCor Pharmaceuticals, Dewpoint, Kowa Pharmaceuticals, Olatec Therapeutics, Medimmune, Novartis, PlaqueTec, and XBiotech, Inc. Dr. Libby's laboratory has received research funding in the last 2 years from Novartis. Dr. Libby is on the Board of Directors of XBiotech, Inc. Dr. Libby has a financial interest in Xbiotech, a company developing therapeutic human antibodies. Dr. Libby's interests were reviewed and are managed by Brigham and Women's Hospital and Partners HealthCare in accordance with their conflict‐of‐interest policies. Dr. Libby receives funding support from the National Heart, Lung, and Blood Institute (1R01HL134892), the American Heart Association (18CSA34080399), the RRM Charitable Fund, and the Simard Fund. Dr. Bhatt discloses the following relationships—Advisory Board: Boehringer Ingelheim, Cardax, CellProthera, Cereno Scientific, Elsevier Practice Update Cardiology, Janssen, Level Ex, Medscape Cardiology, MyoKardia, NirvaMed, Novo Nordisk, PhaseBio, PLx Pharma, Regado Biosciences, Stasys; Board of Directors: Boston VA Research Institute, Society of Cardiovascular Patient Care, TobeSoft; Chair: Inaugural Chair, American Heart Association Quality Oversight Committee; Data Monitoring Committees: Baim Institute for Clinical Research (formerly Harvard Clinical Research Institute, for the PORTICO trial, funded by St. Jude Medical, now Abbott), Boston Scientific (Chair, PEITHO trial), Cleveland Clinic (including for the ExCEED trial, funded by Edwards), Contego Medical (Chair, PERFORMANCE 2), Duke Clinical Research Institute, Mayo Clinic, Mount Sinai School of Medicine (for the ENVISAGE trial, funded by Daiichi Sankyo), Novartis, Population Health Research Institute; Honoraria: American College of Cardiology (Senior Associate Editor, Clinical Trials and News, ACC.org; Chair, ACC Accreditation Oversight Committee), Arnold and Porter law firm (work related to Sanofi/Bristol‐Myers Squibb clopidogrel litigation), Baim Institute for Clinical Research (formerly Harvard Clinical Research Institute; RE‐DUAL PCI clinical trial steering committee funded by Boehringer Ingelheim; AEGIS‐II executive committee funded by CSL Behring), Belvoir Publications (Editor in Chief, Harvard Heart Letter), Canadian Medical and Surgical Knowledge Translation Research Group (clinical trial steering committees), Cowen and Company, Duke Clinical Research Institute (clinical trial steering committees, including for the PRONOUNCE trial, funded by Ferring Pharmaceuticals), HMP Global (Editor in Chief, Journal of Invasive Cardiology), Journal of the American College of Cardiology (Guest Editor; Associate Editor), K2P (Co‐Chair, interdisciplinary curriculum), Level Ex, Medtelligence/ReachMD (CME steering committees), MJH Life Sciences, Piper Sandler, Population Health Research Institute (for the COMPASS operations committee, publications committee, steering committee, and USA national co‐leader, funded by Bayer), Slack Publications (Chief Medical Editor, Cardiology Today's Intervention), Society of Cardiovascular Patient Care (Secretary/Treasurer), WebMD (CME steering committees); Other: Clinical Cardiology (Deputy Editor), NCDR‐ACTION Registry Steering Committee (Chair), VA CART Research and Publications Committee (Chair); Research Funding: Abbott, Afimmune, Amarin, Amgen, AstraZeneca, Bayer, Boehringer Ingelheim, Bristol‐Myers Squibb, Cardax, CellProthera, Cereno Scientific, Chiesi, CSL Behring, Eisai, Ethicon, Faraday Pharmaceuticals, Ferring Pharmaceuticals, Forest Laboratories, Fractyl, Garmin, HLS Therapeutics, Idorsia, Ironwood, Ischemix, Janssen, Javelin, Lexicon, Lilly, Medtronic, MyoKardia, NirvaMed, Novartis, Novo Nordisk, Owkin, Pfizer, PhaseBio, PLx Pharma, Regeneron, Reid Hoffman Foundation, Roche, Sanofi, Stasys, Synaptic, The Medicines Company, 89Bio; Royalties: Elsevier (Editor, Cardiovascular Intervention: A Companion to Braunwald's Heart Disease); Site Co‐Investigator: Abbott, Biotronik, Boston Scientific, CSI, St. Jude Medical (now Abbott), Philips, Svelte; Trustee: American College of Cardiology; Unfunded Research: FlowCo, Merck, Takeda. All remaining authors declare no conflict of interests.

## Supporting information

Supplementary information.

## Data Availability

Requests for access to the data reported in this article will be considered by the corresponding author.
