# Supplementary material for: Early and late recurrent cardiovascular events among high‐risk patients with an acute coronary syndrome: Meta‐analysis of phase III studies and implications on trial design
Source: Clin Cardiol. 2022 Jan 12;45(3):299–307. doi: 10.1002/clc.23773 (PMC8922536; doi:10.1002/clc.23773)

**Table S1.** Characteristics of studies targeting high-risk patients with recent acute coronary syndromes

**Figure S1.** Flow chart of the study selection process

**Figure S2.** Risk of bias assessment

**Figure S3.** Sensitivity analysis of pooled risk at 90 days

**Figure S4.** Sensitivity analysis of pooled risk at 360 days

**Table S1.** Characteristics of studies targeting high-risk patients with recent acute coronary syndromes

| **Study** | **Population** | **Risk factors** | **Follow-up** | **Intervention** | **Concomitant medication** | | | | | **90-day risk** | **360-day risk** |
| --- | --- | --- | --- | --- | --- | --- | --- | --- | --- | --- | --- |
|  |  |  |  |  | **Aspirin** | **P2Y_12_ inhibitor** | **Statin** | **BB** | **ACEI or ARB** |  |  |
| TRITON-TIMI 38 | ACS with scheduled PCI | UA/NSTEMI patients (within 72 hours of onset of symptoms): • TIMI Risk Score for UA/NSTEMI ≥3  STEMI patients: • Primary PCI (within 12 hours of onset of symptoms) • Post-STEMI (>12 hours to 14 days after onset of symptoms) | 15 months | Prasugrel  (N=6,813)  vs.  Clopidogrel  (N=6,795) | 99%  vs.  99% | – | 92%  vs.  92% | 88%  vs.  88% | 76%  vs.  75% | 6.8% vs. 8.5% | 9.3% vs. 11.3% |
| PLATO | Hospitalized for ACS within 1 day | UA/NSTEMI patients with at least one of the following: • Age ≥60 years • Previous MI or CABG • CAD with stenosis of ≥50% in ≥2 vessels • Previous ischemic stroke, TIA, carotid stenosis of ≥50%, or cerebral revascularization • DM • PAD • Chronic renal dysfunction (CrCl <60 mL/min/1.73 m²)  STEMI patients: • Planned primary PCI | 12 months | Ticagrelor  (N=9,333)  vs.  Clopidogrel  (N=9,291) | 97.4%  vs.  97.5% | – | 89.7%  vs.  89.2% | 89.3%  vs.  89.7% | 88.2%  vs.  87.3% | 6.4% vs. 7.4% | 9.8% vs. 11.7% |
| ATLAS ACS 2-TIMI 51 | Hospitalized for ACS within 7 days | Patients aged 18 to 54 years with at least one of the following: • DM • Prior MI | 24 months | Rivaroxaban 2.5 mg  twice daily  (N=5,114)  vs. Rivaroxaban 5 mg  twice daily  (N=5,115)  vs.  Placebo  (N=5,113) | 98.7%  vs.  98.5%  vs.  98.7% | 92.6%  vs.  93.0%  vs.  92.9% | 83.2%  vs.  83.9%  vs.  83.5% | 66.2%  vs.  65.6%  vs.  66.5% | 39.1%  vs.  38.2%  vs.  39.6% | 3.0% vs. 3.1% vs. 3.9% | 6.2% vs. 6.5% vs. 6.8% |
| TRILOGY ACS | Medically managed UA/NSTEMI within 10 days | At least one of the following: • Age ≥60 years • DM • Previous MI • Previous revascularization with PCI or CABG | 30 months | Prasugrel  (N=4,663)  vs.  Clopidogrel  (N=4,663) | 94.0%  vs.  93.4% | – | 83.6%  vs.  83.1% | 78.3%  vs.  77.2% | 75.3%  vs.  75.4% | 3.6% vs. 3.9% | 8.4% vs. 8.6% |
| EXAMINE | ACS within 15 to 90 days | • DM receiving antidiabetic therapy | 18 months | Alogliptin  (N=2,701)  vs.  Placebo  (N=2,679) | 90.6%  vs.  90.8% | 79.8%  vs.  80.8% | 90.6%  vs.  90.3% | 81.7%  vs.  82.2% | 81.5%  vs.  82.5% | 3.3% vs. 3.4% | 8.9% vs. 9.1% |
| AleCardio | Hospitalized for ACS within 8 weeks | • Established (managed by diet or medication) or newly diagnosed DM | 24 months | Aleglitazar  (N=3,616)  vs.  Placebo  (N=3,610) | 95.6%  vs.  95.3% | 88.9%  vs.  87.3% | 92.1%  vs.  93.0% | 83.5%  vs.  83.7% | 82.6%  vs.  82.1% | 2.7% vs. 2.7% | 6.9% vs. 6.7% |
| SOLID-TIMI 52 | Hospitalized for ACS within 30 days | At least one of the following: • Age ≥60 years • History of MI • Significant renal dysfunction (eGFR 30–59 mL/min/1.73 m²) • DM requiring pharmacotherapy • Polyvascular disease (including carotid or PAD) | 36 months | Darapladib  (N=6,504)  vs.  Placebo  (N=6,522) | 96.4%  vs.  96.5% | 88.3%  vs.  88.4% | 94.3%  vs.  94.9% | 87.2%  vs.  87.4% | 82.7%  vs.  82.4% | 3.2% vs. 3.3% | 7.5% vs. 7.3% |

***Abbreviations:*** ACEI, angiotensin-converting enzyme inhibitor; ACS, acute coronary syndrome; ARB, angiotensin II receptor blocker; BB, beta-blocker; CABG, coronary artery bypass grafting; CAD, coronary artery disease; CrCl, creatinine clearance; DM, diabetes mellitus; eGFR, estimated glomerular filtration rate; MI, myocardial infarction; PAD, peripheral artery disease; PCI, percutaneous coronary intervention; STEMI, ST-segment elevation myocardial infarction; TIA, transient ischemic attack; TIMI, thrombolysis in myocardial infarction; UA, unstable angina.

**Figure S1.** Flow chart of the study selection process


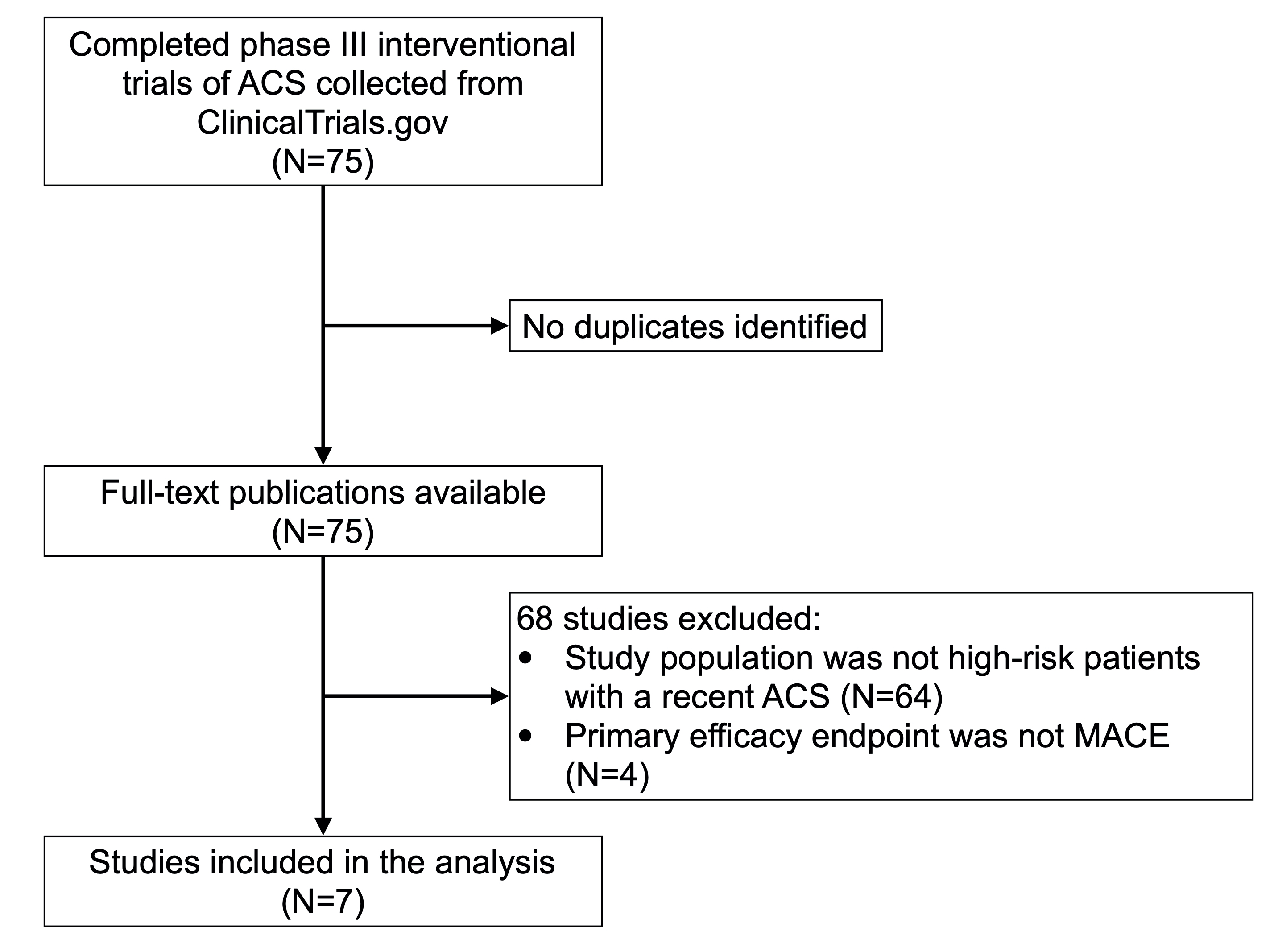


**Figure S2.** Risk of bias assessment


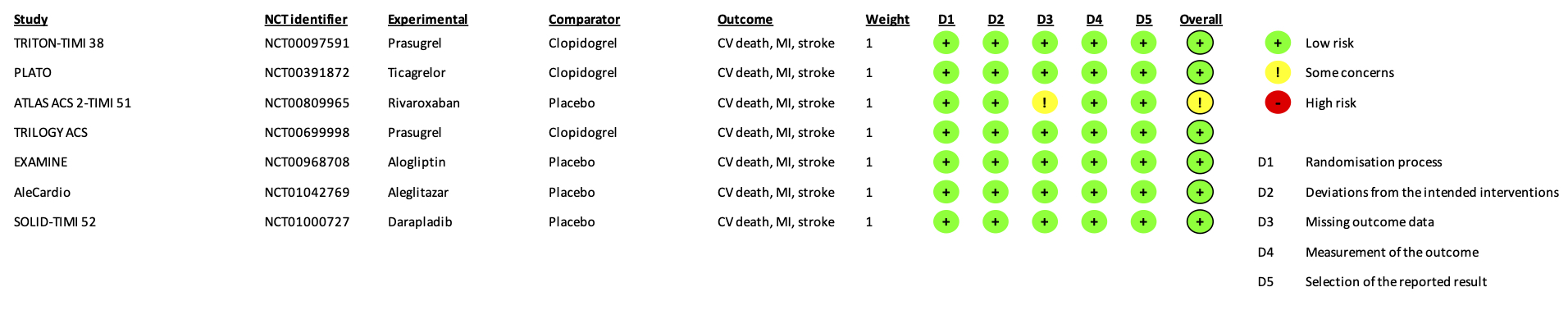


**Figure S3.** Sensitivity analysis of pooled risk at 90 days


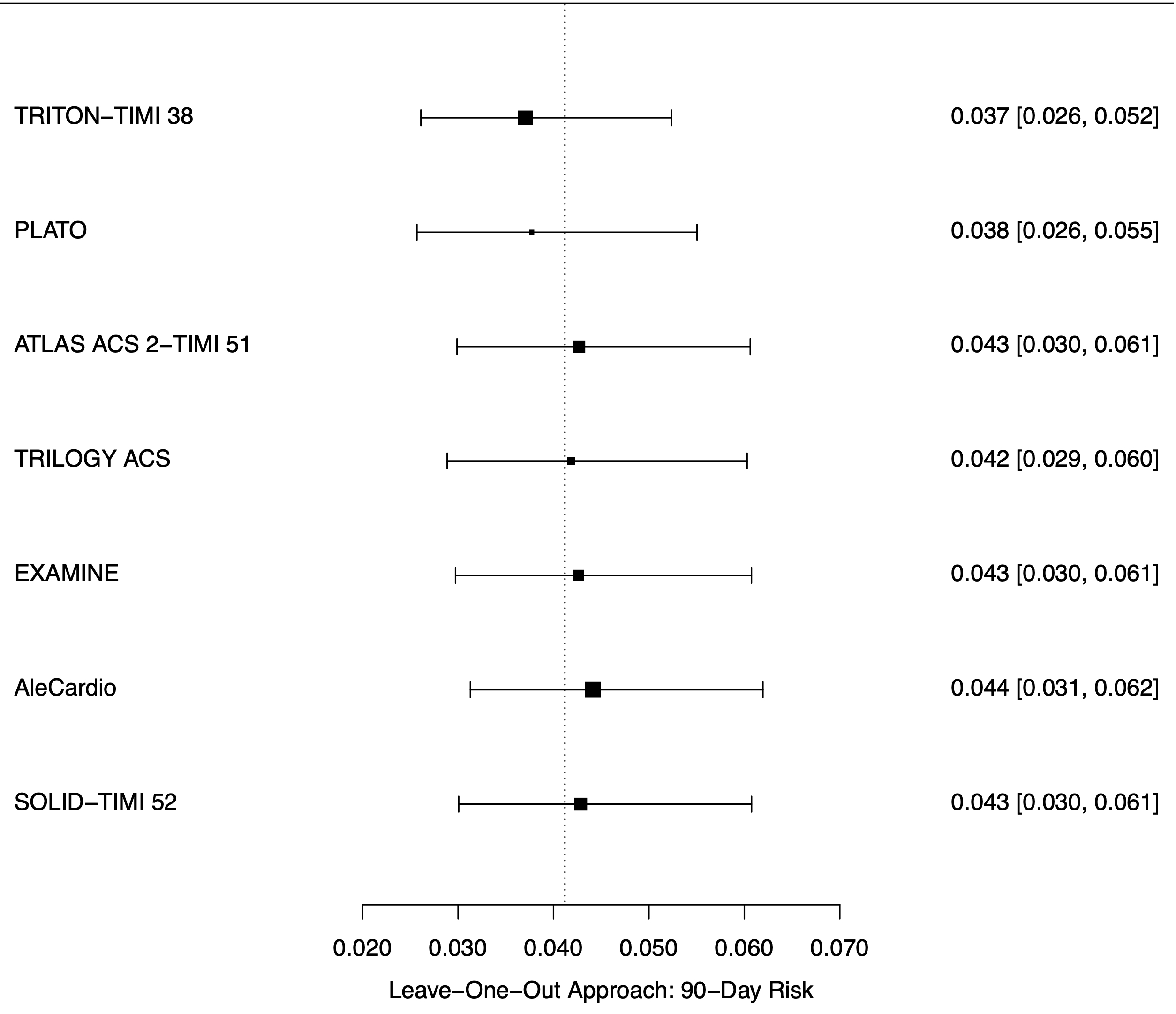


**Figure S4.** Sensitivity analysis of pooled risk at 360 days


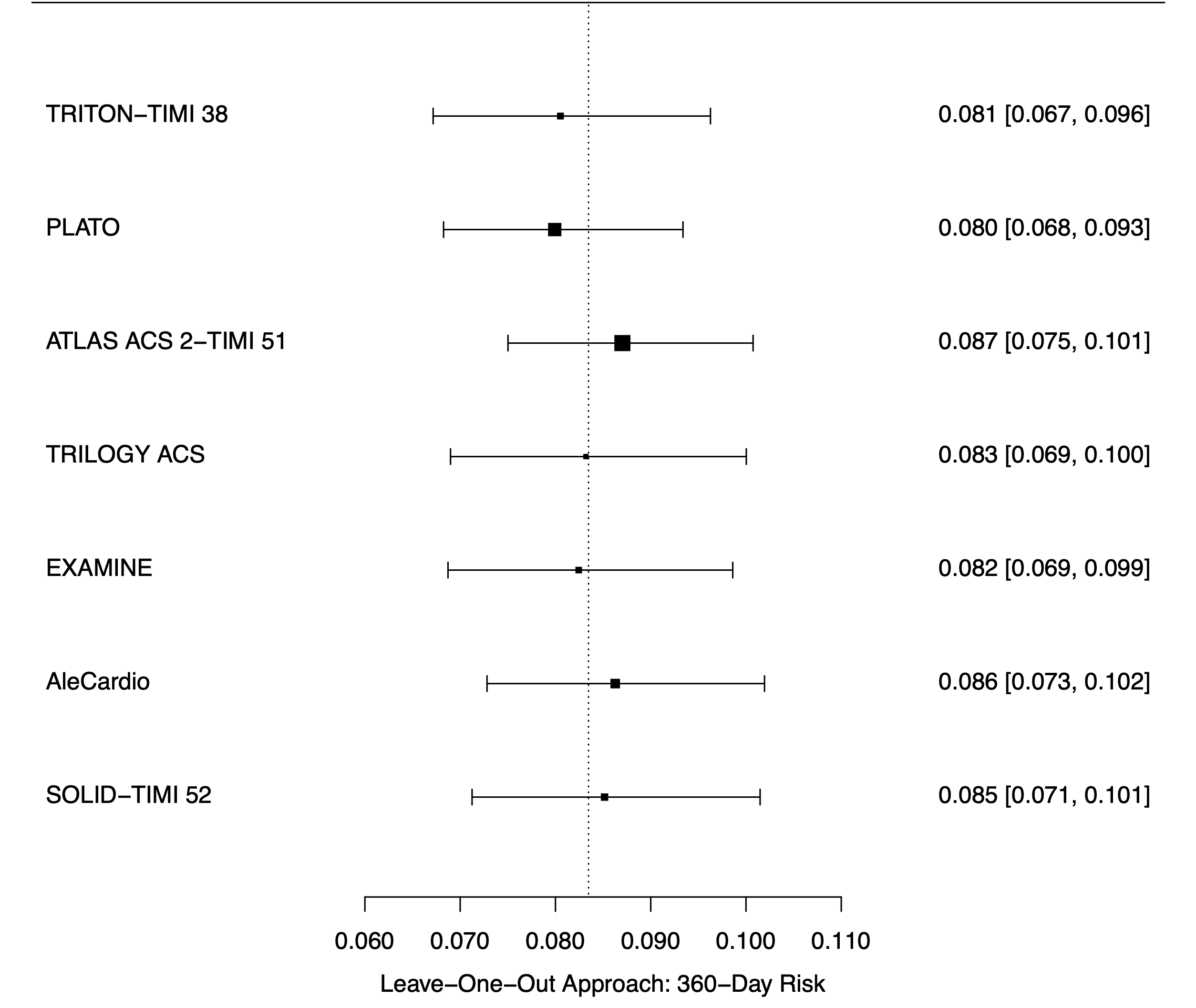

Supplement: Supplementary file 1 — Supplementary information. [file CLC-45-299-s001.docx]
